# Wage gains from foreign ownership: evidence from linked employer–employee data

**DOI:** 10.1186/s12651-021-00286-0

**Published:** 2021-02-15

**Authors:** János Köllő, István Boza, László Balázsi

**Affiliations:** 1 Institute of Economics, Hungarian Academy of Sciences, Budapest, Hungary; 2 Central European University, Budapest, Hungary

**Keywords:** Multinational enterprises, FDI, Wage differentials, Wage spillover, Hungary, F23, J24, J31, O33

## Abstract

We compare the wages of skilled workers in multinational enterprises (MNEs) versus domestic firms, the earnings of domestic firm workers with past, future and no MNE experience, and estimate how the presence of ex-MNE peers affects the wages of domestic firm employees. The analysis relies on monthly panel data covering half of the Hungarian population and their employers in 2003–2011. We identify the returns to MNE experience from changes of ownership, wages paid by new firms of different ownership, and the movement of workers between enterprises. We find high contemporaneous and lagged returns to MNE experience and significant spillover effects. Foreign acquisition has a moderate wage impact, but there is a wide gap between new MNEs and domestic firms. The findings, taken together, suggest that MNE employees accumulate partly transferable knowledge, valued in the high-wage segment of the local economy that is connected with the MNEs via worker turnover.

## Introduction

While policymakers in developing countries are often criticized for ‘selling out’ the country to foreigners, FDI can actually bring valuable knowledge to a less developed economy, spreading through labor mobility channels. Undeniably, corporate revenues can find their way back home via profit repatriation and transfer pricing, and many MNEs enjoy a generous initial tax holiday. However, MNE workers’ wage premium over similar domestic-sector employees in comparable firms directly benefits society, especially if the underlying excess productivity is portable and exerts positive spillover effects. Unlike the returns to capital investment and part of the profit, the wage surplus predominantly remains and is spent in the host country.

The literature provides ample evidence to call into question the *general* validity of such an optimistic scenario. The foreign-domestic wage gap is negligible in countries close to the productivity frontier (Balsvik 2011; Heyman et al. 2007; Andrews et al. 2007; Malchow-Moller et al. 2007). An adverse competition effect often offsets the positive direct impact of FDI on productivity and wages even in relatively undeveloped economies (Aitken and Harrison 1999; Djankov and Hoekman 2000; Konings 2001; Barry et al. 2005). The positive spillovers are often restricted to specific sectors (Keller and Yeaple 2009; Suyanto and Bloch 2014; Fons-Rosen et al. 2017). Still, the existence of a vast MNE premium in the emerging and transition economies (Lipsey and Sjöholm 2004; OECD 2008a; Chen et al. 2017), and the findings of positive spillovers (Smarzynska-Javorcik 2004; Görg and Strobl 2005; Kosová 2010; Poole 2013; Gorodnichenko et al. 2014) encourage us to seek evidence for a ‘knowledge flows’ scenario.^1^ To assess the magnitude of the potentially beneficial impact of FDI, we study the direct and indirect wage effects of work experience in multinational enterprises (MNEs) using linked employer–employee data on skilled workers in Hungary, 2003–2011.

We contribute to the literature by empirically showing in a single study that (i) MNEs pay markedly higher wages than similar domestic firms. (ii) MNE employees lose a part of their wage advantage upon leaving the foreign-owned sector. (iii) Even so, they earn more than their colleagues in domestic enterprises. (iv) Domestic firm employees benefit from having ex-MNE peers. We interpret the *coincidence* of the MNE premium, partial wage loss from separation, lagged returns to MNE experience, and wage spillovers as a signal of knowledge transfer from MNEs to domestic firms. While alternative explanations exist for each of the presented symptoms,^2^ in the last section of the paper we argue that a ‘knowledge flows’ scenario has the best chance to produce *all* of the four outcomes.

Regarding methodology, we draw attention to the difficulties of identification coming from the non-random selection of firms into foreign ownership and of differently skilled workers into foreign enterprises. We find trade-offs between model quality and unbiasedness of the samples on which the first-best models can be estimated.

The analysis is based on a big administrative panel data set covering half of the Hungarian population and their employers in 2003–2011. We restrict the analysis to skilled workers for three reasons.^3^ First, the traces of knowledge transfer are easier to find in the skilled labor market. Second, data discussed later suggest that a part of the MNE premium compensates unskilled workers for non-wage disamenities like shift work, weekend work, and a higher probability of becoming unemployed. The data does not indicate ownership-specific differences of this kind among highly skilled workers. Third, repeating the estimations for middling and unskilled workers would triple the statistics to be presented, with minimal added content. Estimation on a pooled sample would only attenuate the relevant parameters.

We start by estimating the *foreign-domestic wage gap* using panel regressions. By gradually removing the effects of observed and unobserved worker and firm characteristics, we get from a substantial raw gap of 0.75 log points to 0.24 points after controlling for worker fixed effects and a mere 0.03 points’ pure ownership-specific wage differential estimated with both worker and firm fixed effects (2FE henceforth).

While a 2FE model can answer how an existing firm’s wage level changes in response to a change in ownership, the effect it identifies is unsuitable for out-of-sample prediction. Only 5.3 percent of the observed firms changed the majority owner during the observation period in our sample. These companies paid significantly higher wages than ‘always domestic’ firms (when they were domestic) and significantly lower wages than ‘always foreign’ companies (when they were foreign-owned): this is how the 2FE model arrives at a close-to-zero estimate of the ownership-specific wage gap. These firms’ experience can hardly predict how big MNEs like Mercedes-Benz or IBM would pay their employees in the unlikely event of takeover by a local business person. It also tells nothing about the potential wage gains from greenfield investments, which played a significant role in the 1990s (Calderon et al. 2004).^4^ We utilize a difference-in-difference estimation of wage gains from joining a new MNE over joining a new domestic firm to learn about the ownership-specific wage gap between ‘always foreign’ and ‘always domestic’ companies. This approach suggests that the employees of new MNEs earn 15 percent more than their domestic counterparts.

Turning to the *MNE premium’s portability*, we have to deal with endogeneity and ability biases, as worker mobility is not random. If a worker is fired from an MNE, it may be because her marginal product is lower than average. If a domestic employer attracts a worker, it may be because she has a higher-than-average marginal product irrespective of the sector of employment. To address the first problem, we compare domestic firm employees with recent MNE experience to their peers who had outside experience in the domestic sector. We focus on workers losing or leaving their jobs in times of mass dismissals when separations are more likely to be exogenous to the individual worker’s productivity. The model controls for heterogeneity of the sending firms via observable controls and use fixed effects for the receiving ones. We find that former MNE employees earn more by 13 percent than similar workers coming from collapsing domestic enterprises. Workers separating from their employers for reasons other than mass dismissals acquire a significantly lower (5 percent) lagged MNE premium.

Satisfactory model quality comes at the cost of distortions in the sample and a significant loss of observations in this case, too. Only about 7 percent of the person-months in our data makes it to the estimation sample of a model in which work histories and characteristics of the sending and receiving firms are adequately controlled. The problem would be further aggravated by the inclusion of worker fixed effects to reduce ability bias.^5^ To avoid this issue while utilizing more data and still controlling for the potential bias, we rely on a less demanding ‘overlapping cohorts’ model that compares domestic firm employees with future and past experience in foreign versus domestic firms. This model can utilize a much broader sample, as workers with only two observed spells can contribute to the estimation if any of those is at a foreign-owned employer. The estimated return to prior MNE experience amounts to 0.07 log points.^6^

Finally, we estimate *spillover effects* for incumbent domestic firm employees, controlling for observed and unobserved worker and firm characteristics. We deviate from a similar attempt by Poole (2013) in two ways. First, we also study how skilled incumbents’ wages respond to the presence of less qualified ex-MNE peers. Second, and more importantly, we address the selection problem that arises when the analysis is restricted to incumbents (domestic workers with no experience outside their firms). Incumbents in our data account for only 22 percent of the workers ever employed in the domestic sector. Their exposure to peers with MNE experience differs substantially from that of the average worker. In an alternative specification, we ensure the identification of within-firm spillovers using a 2FE model. We find that a one-standard-deviation difference in the share of high skilled ex-MNE peers shifts peers’ wages with no MNE past up by slightly more than one percent. Having qualified peers with outside experience in the domestic sector and having low-skilled peers with MNE experience do not affect wages.

Section 2 discusses previous findings on the paper’s topic and prewarns the reader of our estimates. Section 3 introduces the data and the local context. Section 4 is devoted to the study of the foreign-domestic wage gap. Sections 5 and 6 present the results on lagged returns and spillover effects, respectively. Section 7 briefly comments on differences by skill levels and industries. Section 8 sums up the results and argues that the empirical findings, taken together, yield support to a ‘skills diffusion’ scenario.

## Previous findings on the foreign-domestic wage gap, lagged returns and spillovers

Estimates of the *foreign-domestic wage gap* vary widely, with the MNE premium found to be nearly negligible in the most developed market economies. In Norway, the OLS estimate by Balsvik (2011), controlled for worker and plant characteristics, amounts to 3 percent, which falls to 0.3 percent once she includes worker fixed effects. An OLS estimate for Sweden by Heyman et al. (2007) is even lower at 2 percent. Andrews et al. (2007) and Malchow-Moller et al. (2007) detect positive gaps in the range of 1 and 3 percent in Germany and Denmark. The OLS estimate of Martins (2004) for Portugal is higher (11 percent), but he finds that the MNE wage premium virtually disappears after controlling for worker selection. These figures compare to 32 percent (pooled OLS for all skill levels) and 13 percent (after adding worker fixed effects) in our sample. Workers moving from domestic to foreign-owned firms are estimated to gain 6 percent in Germany and 8 percent in Norway (Andrews et al. 2007; Balsvik 2011), which compares to 53 percent in the Hungarian sample for all skill levels.^7^

The foreign-domestic gap is much broader in less developed countries: according to raw data presented in Lipsey and Sjöholm (2004), in Indonesian manufacturing, the MNE premium amounts to 47 percent for blue collars and 55 percent for white collars (41 and 73 percent in Hungary). Chen et al. (2017) report a gap of 40 percent in Chinese manufacturing. An overview of data in OECD (2008a), based on the World Bank Enterprise Survey, indicates raw gaps of between 40 and 50 percent in Africa, Asia, the Middle East, and combining all these regions and adding Central and Eastern Europe.

A more detailed analysis of the sources of the gaps in Germany, Portugal, the UK, and Brazil (OECD 2008b) finds that takeovers’ marginal effect on wages falls short of 3 percent in all of these countries.^8^ Results from Hungary point to similar patterns. Csengődi et al. (2008) use a different data set from ours (the Wage Survey, a repeated cross-section LEED which allows the linking of firms but not workers) and find that after adding firm fixed effects, the MNE wage premium falls to a mere 3 percent as it does in our case.^9^ Earle et al. (2017) use the same data source and detect a slightly higher premium of 7 percent that is still very far from the estimates they get without controlling for unobserved firm characteristics and firm-specific trends. The effects identified using data on worker mobility by OECD (2008b) are more substantial: the estimates vary between 6 and 8 percent in Germany and the UK, more than 10 percent in Portugal, and 20 percent in Brazil. The authors argue that the discrepancy between the estimates based on takeovers versus worker flows are explained by foreign firms’ propensity to share their productivity advantage more extensively with new workers than with workers who do not change firms. We believe that the difference instead roots in the non-random selection of firms to acquisition, as will be discussed in more detail later.

To our knowledge, Balsvik’s paper is the only one estimating the *wage advantage of ex-MNE employees in domestic firms*. She identifies a premium of 6.9 percent for workers with three or more years of tenure in an MNE. However, she also detects an advantage of 3.3 percent on the part of workers arriving from local firms, suggesting a net benefit from MNE experience of 3.6 percent (and smaller advantages in case of shorter completed tenure in the previous job). We find that domestic firm employees, who left an MNE because of mass dismissals, closure, or relocation earn more than their ex-domestic counterparts by 13 percent.

The empirical evidence on wage and productivity spillovers are mixed. Starting with papers that depict a not too rosy picture of how MNEs affect the rest of the economy, Aitken and Harrison (1999) and Djankov and Hoekman (2000) identify a positive direct effect of foreign ownership on productivity in Venezuela and the Czech Republic, but negative spillovers. Konings (2001) suggests that the adverse competition effect is stronger than the positive direct productivity effect of FDI in Bulgaria, Romania, and Poland. Barry et al. (2005) found that foreign presence in a sector hurts wages and productivity in domestic exporting firms in the same industry (but does not affect wages in domestic non-exporters) in Ireland. Fons-Rosen et al. (2017) conclude that in six advanced European countries, positive spillovers are restricted to sectors where domestic enterprises are technologically close to MNEs. Suyanto and Bloch (2014) find the opposite in Indonesia. Keller and Yeaple (2009) detect significant worker-level wage spillovers only in high-skill-intensive industries in US manufacturing. By looking at existing firms in an Audi plant’s supplier industries in Hungary, Bisztray (2016) finds no positive effect on productivity. She also finds that firms with foreign owners account for all the positive impact on sales and employment, suggesting a foreign-to-foreign complementarity rather than a galvanizing effect on the domestic sector.

At the same time, several studies have identified positive spillovers. Using Lithuanian data, Smarzynska-Javorcik (2004) detects positive productivity spillovers from MNEs to local suppliers. Similarly, Gorodnichenko et al. (2014) find that backward linkages positively affect the productivity of domestic firms (while horizontal and forward linkages show no consistent effect) in 17 transition countries. Using Czech data, Kosová (2010) demonstrates that crowding out is short-term: after an initial shock, domestic firm growth accelerates, and survival rates improve. Görg and Strobl (2005) show that entrepreneurs with MNE experience start more productive small businesses in Ghana. Bisztray (2016) found that new entrants’ growth in productivity was significantly higher when located close to Audi and operated in a supplier industry.

Importantly, from this paper’s point of view, Poole (2013) estimates that the wages of incumbent domestic firm employees in Brazil rise by about 0.6 percent if the share of ex-MNE employees increases by 10 percent, while the effect of outside experience in local firms is about ten times weaker than that. While the effect she estimates is not particularly strong, it is statistically significant at conventional levels.

One can also find indirect evidence on spillovers, considering that MNEs are more productive and more likely to export and engage in R&D. Stoyanov and Zubanov (2012) show that (in Denmark) workers coming from more productive firms experience productivity gains. Similar results are presented for Hungary by Csáfordi et al. (2018). Mion and Opromolla (2013) show that export experience implies higher export performance and a sizable wage premium for Portuguese managers, who leave for non-exporters. In Finland, Maliranta et al. (2008) identify positive impact of hiring workers with previous R&D experience to non-R&D jobs.

## Data and the local context

### Data sources

Our estimation samples have been drawn from a big longitudinal data set covering a randomly chosen 50 percent of Hungary’s population aged 5–74 in January 2003. Each person in the sample is followed monthly, from January 2003 until December 2011, or exit from the registers for death or permanent out-migration. The data collect information from the Pension Directorate, the Tax Office, the Health Insurance Fund, the Office of Education, and the Public Employment Service. We use information on the highest paying job of a given person in a given month, days in work, and amounts earned in that job. Throughout the paper, we use daily wages (the monthly value divided by days in work) normalized for the given month’s national average. We have data on occupation, type of employment relationship, registration at a labor office, receipt of transfers, and several proxies of the person’s state of health. We do not observe educational attainment—this is approximated with the person’s highest occupational status in 2003–2011.^10^ The data on firms come from the annual tax reports of businesses obliged to conduct double book-keeping. The firm-level variables are merged into the respective person-month observations. We regard a firm as MNE if foreigners’ share in subscribed capital exceeds 50 percent.^11^

We restrict the analysis to skilled workers employed at least once in a foreign or domestic private enterprise the employment level of which exceeded the ten workers limit at least once in 2003–2011. We have several reasons to set a size limit. First, foreign firms are nearly absent in the small firm sector.^12^ Second, financial data are not available for sole proprietorships and unincorporated small businesses. Third, the financial reports of incorporated small firms are often incomplete and erroneous. Finally, the earnings data of small firms are flawed by paying “disguised” minimum wages.^13^ Small firms’ inclusion would also raise the risk of measurement error in the analysis of spillover effects since the probability of not observing an ex-MNE employee in a 50-percent sample is much higher in small establishments. We iteratively removed workers and firms with less than two data points, zero wages, and missing covariates.

After these steps of data cleaning, we are left with a sample of 19,961,622 person-months belonging to 344,203 skilled workers and 119,580 firms. 52.6 percent of the workers had at least one spell of employment in the foreign-owned sector, of which 21.5 percent worked only in MNEs. We draw special sub-samples from this starting population for the study of new firms, lagged returns and spillover effects. Descriptive statistics are presented in Table 11 of Appendix 1.

Even though our firm-level variables are of annual frequency, we prefer to analyze the data at a monthly level for several reasons. First, the affiliation of a worker cannot be precisely measured on a yearly basis. About 25 percent of the workers employed by an MNE for at least one month in a given year also had one or more spells in the domestic sector in the same year. Second, turning to a yearly basis would impair the precise measurement of tenure and the time between two jobs—essential controls in the analysis of lagged returns. Third, higher observed mobility helps in identifying firm and person effects. The problem raised by inflating observations at the same firm is taken care of by the worker *and* firm-level clustering of errors.

### MNEs in Hungary

In the first decade after the start of the transition, Hungary was the most successful country within the former Soviet bloc in attracting foreign capital. By 2003, the beginning of our observation period, cumulative FDI inflows exceeded 40 percent of the GDP,^14^ multinationals employed 15 percent of the labor force (including self-employment and the public sector into the denominator) and more than 30 percent of private-sector employees. They produced 20 percent of the GDP and delivered over two-thirds of the exports (Balatoni and Pitz 2012). Large multinationals, including Audi, General Motors, and Suzuki, dominated the motor industry. Foreign presence was already significant in the tobacco, leather, chemical, rubber, and electronics industries, with employment shares of between 50 and 80 percent.

Almost three-fourths of the cumulative FDI inflows have arrived in sectors outside of manufacturing. As shown in column 4 of Table 1, nearly 60 percent of the skilled employees within the MNE sector worked in the tertiary sector. Therefore, we do not restrict the analysis to manufacturing, as most papers do in the strand of the literature we follow (see Barry et al. 2005; Görg and Strobl 2005; Lipsey and Sjöholm 2004; Smarzynska-Javorcik 2004; Balsvik 2011 as opposed to Poole 2013, whose study covers all sectors in Brazil). While FDI typically boosts exports and generates demand for domestic manufacturers producing intermediate goods, its contribution to the quality of retail trade, banking and services can be equally important, especially in the former state-socialist countries, which started the transition with critically undeveloped non-tradable sectors. The foreign-owned and domestic parts of the economy are closely connected via labor turnover. In the skilled labor market, 37.2 percent of the domestic firms, employing 69 percent of the domestic labor force, hired at least one ex-MNE worker in 2003–2011.

**Table 1:** Foreign ownership in Hungary, 2003

	Fraction employed in MNEs (percent of all person-months in the given industry)		Industrial composition of MNEs (percent of all person-months in the MNE sector)	
	All workers	Skilled workers	All workers	Skilled workers
Agriculture	5.0	6.1	0.8	0.5
Manufacturing	46.5	48.4	59.9	40.5
Construction	7.7	10.6	1.5	1.9
Energy, water, gas	57.5	55.6	3.3	3.1
Wholesale and retail trade	25.9	34.5	16.3	31.5
Finance and insurance	52.7	80.0	11.4	11.5
Services	20.7	24.3	6.8	11.0
Average/total	34.8	37.6	100.0	100.0

The data are annual averages observed in the estimation sample in 2003. The number of person-months amount to 8,704,486 (all workers) and 2,068,556 (skilled workers)

### Descriptive statistics on wages and wage change

Table 2 presents raw statistics on wage levels across ownership categories and wage changes associated with skilled workers’ shifts between them. The data shows vast differences between workers in MNEs versus domestic firms, on the one hand, and domestic firm employees hired from MNEs versus workers coming from other domestic enterprises, on the other.

**Table 2:** Descriptive statistics: wage levels and wage changes of skilled workers

	Mean	St. dev.	Observations
Wage levels^a^			
Employer = MNE	309	288	7,937,675^b^
Employer = domestic firm	143	161	12,023,947^b^
Wage change upon leaving an MNE for a domestic firm^c^			
Mean	− 57	146	42,479
Median	− 26		42,479
Wage change upon leaving a domestic firm for an MNE^c^			
Mean	64	126	46,590
Median	39		46,590
Wages of domestic firm employees with recent outside experience^d^			
Previous employer = MNE	171	193	963,075^a^
Previous employer = domestic firm	118	122	3,557,788^a^

^a^Wage in month *t* relative to the national average wage in month *t*, per cent

^b^Person-months observed in 2003–2011

^c^The figures relate to persons moving from MNEs to domestic firms and vice versa. Mean earnings in the receiving firm is compared to the same worker’s mean earnings in the sending firm. Wages are deflated with the national average wage in the same month

^d^The figures relate the mean earnings of domestic firm employees with previous outside experience to the mean earnings of incumbent domestic firm employees, percent

According to the raw data, MNE employees earn more than twice as much as domestic sector workers. Persons moving from domestic firms to MNEs gain 64 percentage points on average, while individuals who move to the other direction lose 57 points. Measured with the median rather than the mean, the gain and the loss amount to 39 and − 26 percentage points, respectively.^15^ The bottom block suggests a substantial raw premium for outside experience in foreign-owned enterprises. In the forthcoming sections, we try to disentangle a ‘pure’ ownership-specific effect from differences in composition.

## Foreign-domestic wage gap

### Benchmark model

Our first model estimates the foreign-domestic wage gap in the following way:

1 $$\ln w_{ijt} =\, \delta F_{ijt} + \left[ {\varphi P_{i} } \right] + \alpha X_{it} + \beta Y_{ijt } + \gamma V_{jt} + \left[ {v_{i} + f_{j} } \right] + s_{jt} + \varepsilon_{ijt} ,$$

where $w_{ijt}$ is the daily average (relative) earnings of person $i$ at firm $j$ and month $t$, $F$ is a dummy for being employed in a majority foreign-owned firm, $P_{i}$ and $X_{it}$ are fixed and time-varying individual attributes, *Y*_*ijt*_ stands for job-specific variables (like occupation and tenure), $V_{jt}$ denotes time-varying firm-specific covariates, $v_{i}$ and $f_{j}$ are worker and firm fixed effects, respectively, and ε_ijt_ is an error term. We allow for unobserved shocks to productivity by including sector–year interactions $s_{jt}$. The firm-level variables are size, the capital-labor ratio, and a dummy for exporters. Alternatively, we use indicators of investment and productivity. We gradually move from an OLS equation only controlled for $s_{jt}$ to fixed-effects models with all the covariates except for the $P_{i}$ variables.

When the equation is estimated with OLS, the δ parameter captures the ownership effect, plus the employment-duration weighted average residual *worker* and *firm* effects given personal characteristics $P$ and $X$ (Abowd et al. 2006). The person fixed effects absorb the unobserved time-invariant mean “qualities” of workers. However, the estimated gap is still affected by the employment-duration weighted average of the firm effects for the firms in which the worker was employed. When both person and firm fixed effects are included, δ captures a pure ownership effect identified from worker flows between ownership categories, on the one hand, and changes in ownership, on the other.^16^ It shows the wage advantage of a foreign firm employee over a domestic worker with similar observable attributes, controlled for their average wages in the entire period of observation, and also controlled for average wages of the firms where they worked during the period of observation. For our multiple fixed effect estimations, we use a model proposed by Correia (2017), and implemented in Stata as *reghdfe*.^17^

In Model A of Table 3, which measures MNE employees' wage advantage relative to domestic firm employees, the estimate rises from 0.745 log points to 0.763 after controlling for observed worker characteristics. The inclusion of firm size, the capital-labor ratio, and exports bring the estimated MNE premium down to 0.437, while adding worker fixed effects reduces it to 0.236. Adding firm fixed effects results in a major drop to only 0.026.

**Table 3:** Estimates of the foreign-domestic wage gap for skilled workers, 2003–2011

Specifications:	(1)	(2)	(3)	(4)	(5)	(6)
Model A						
Foreign-owned	0.745 (25.5)	0.763 (20.3)	0.718 (31.6)	0.437 (23.4)	0.236 (28.6)	0.026 (3.5)
aR2/within R2	0.260	0.329	0.414	0.480	0.238	0.103
Model B						
Always foreign-owned	0.794 (25.7)	0.817 (29.5)	0.772 (31.3)	0.507 (23.3)	0.307 (26.8)	
Temporarily foreign-owned	0.569 (8.1)	0.574 (8.7)	0.564 (12.6)	0.334 (5.2)	0.209 (14.1)	0.026 (3.5)
Temporarily domestic	0.408 (10.5)	0.408 (10.5)	0.462 (11.3)	0.269 (8.7)	0.157 (11.6)	
aR2/within R2	0.267	0.327	0.422	0.482	0.242	0.103
Controls						
Sector × year	Yes	Yes	Yes	Yes	Yes	Yes
Person	No	Yes	Yes	Yes	Yes	Yes
Job	No	No	Yes	Yes	Yes	Yes
Firm	No	No	No	Yes	Yes	Yes
Person FE	No	No	No	No	Yes	Yes
Firm FE	No	No	No	No	No	Yes

All coefficients are significant at 0.01 level, t-values in parentheses. The standard errors are adjusted for clustering by persons and firms. Sample: 19,961,622 person-months belonging to 344,203 skilled workers in 119,580 firms. Singleton observations are excluded from the panel regressions Dependent variable: log daily wage in the given month relative to the national mean. Reference categories: employed in a domestic firm (Model A), employed in an’ always domestic’ firm (Model B). Controls: person, job and firm characteristics plus sector–year interactions. See Appendix 1: Table 12 for variable definitions. Specifications 5 and 6 include only time-varying covariates and worker and firm fixed effects. Estimation: all models were estimated with Stata’s *reghdfe* models

Controlling the worker fixed-effect model for TFP or value-added per worker instead of the firm fixed effects yield estimates of 0.218 and 0.206, respectively. Including TFP into the set of firm controls in specification (4) results in a coefficient of 0.209. Including investment as well, which controls for the potential coincidence of positive productivity shocks and the hiring of high-quality labor, produces an estimate of 0.216. By contrast, adding firm fixed effects to specification (4) without including worker fixed effects decreases the estimate from 0.437 to 0.036, clearly indicating that selection to acquisition drives the result of the 2FE model.

In Model B of Table 3, the observed person-months are classified by the ownership histories of employers. ‘Always domestic’ (the reference category) and ‘always foreign’ denote enterprises that did not change owner in 2003–2011. ‘Temporarily foreign’ and ‘temporarily domestic’ indicate the current majority owner of the workers’ employer, for firms which underwent acquisition at least once in 2003–2011. The ‘temporary foreign’ dummy, for instance, is set to one for a person-month spent in a foreign-owned enterprise, which operated under domestic ownership in a part of the observed period.^18^

The estimates suggest that firms involved in takeovers and currently operating under domestic ownership pay more than incumbent domestic firms (by 0.157 log points in specification 5 where worker quality is controlled for). Switching firms currently under foreign ownership pay lower wages than always foreign-owned companies by 0.099 log points. The gap between the coefficients for employment spells under ‘temporarily foreign’ and ‘temporarily domestic’ ownership (0.052 log points) is an alternative measure of how ownership changes affect the wage. The magnitudes make it clear that switching firms substantially differ from any of the incumbent categories.

### Exploiting information on new firms

As much as 94.8 percent of the firms in our estimation sample did not change majority owner in the period covered by the data: 7.3 percent was foreign-owned, and 87.5 percent was domestic throughout 2003–2011. Rather than merely neglecting the huge wage difference between them (as does the 2FE model), we exploit information on newly established and subsequently incumbent foreign and domestic enterprises. The critical event under examination here is not the takeover of an existing firm, but the birth of an incumbent firm. The analysis relates to firms established after 2003 and staying under majority foreign or domestic control until 2011. We compare the earnings of incumbent workers in these firms to the wages they earned before their entry. Formally, we estimate the following difference-in-difference model:

2 $$\ln w_{ijt} = \beta_{1} F_{ijt}^{0} + \beta_{2} D_{ijt}^{0} + \beta_{3} F_{ijt}^{1} + \beta_{4} D_{ijt}^{1} + \user2{Z\gamma } + \varepsilon_{ijt} .$$

F and D are the acronyms for foreign-owned and domestic firms. F^0^ and D^0^ are set to one for person-months preceding the worker’s entry date to a newly established F or D firm. F^1^ and D^1^ are set to one for the months of service in a newly established firm. For instance, for a worker hired by a new foreign-owned company in month t = 37, F^0^ = 1 if t < 37 and F^1^ = 1 if t ≥ 37. **Z** denotes controls listed in the notes to Table 6.

β_1_ − β_2_ is the estimated wage difference between *future* F and D employees, whereas β_3_ − β_4_ measures the wage difference between the employees of new F and D firms. The double difference (β_3_ − β_4_) − (β_1_ − β_2_) removes the gap in the quality of F and D workers as measured with their pre-entry wages. Since assignment to the groups compared is person-specific, and the firms do not change owner, we estimate the equation with pooled OLS. A large battery of controls guarantees that we compare workers and firms with similar characteristics.

Note that we base the definition of a ‘new firm’ on its employment dynamics rather than its date of registration since the latter is often associated with break-ups, mergers and acquisitions, rather than the birth of a new economic actor. We rely on the fact that a medium-sized or large firm’s creation typically begins with hiring a small group of managers who arrange the start-up. This preparatory stage is followed by a ‘big bang’ when the firm hires rank-and-file employees. We speak of a big bang when a firm’s staff jumps from an initial level of $L_{t - 1}$ ≤ 5 to $L_{t}$ ≥ 50, or, from $L_{t - 1}$ ≤ 50 to $L_{t}$ ≥ 300 within a month. We found 519 such firms with no subsequent change of ownership. Combined employment in these enterprises jumped from 13 to 253 thousand (see Appendix 1: Fig. 3). Finally, the sample consists of 471,489 person-months belonging to 8225 skilled workers hired by and staying until December 2011 in 366 new domestic and 147 new foreign-owned firms.

The results in Table 4 indicate a wage gap of 0.391 log points between skilled workers in new MNEs versus new domestic firms—this is reasonably close to the 0.437 log points gap estimated with a fully controlled OLS for all firms in Table 3, specification 4. New foreign firms' workers also earned more than their domestic counterparts before they entered the new firms by 0.245 log points on average. After deducting this difference from the post-entry gap, an ownership-specific wage differential of 0.146 log points remains between incumbent workers in incumbent firms. This point estimate falls between the individual only and the two fixed-effects parameters, suggesting a significantly stronger pure ownership-specific effect than the 2FE model.

**Table 4:** Wages before and after entry to new MNEs and new domestic firms

	Coeff.	t-test	Person-months
Workers of domestic start-ups, before their entry	0		115,443
Workers of foreign start-ups, before their entry	0.245***	8.3	146,585
Workers of domestic start-ups, after their entry	− 0.014	0.3	84,018
Workers of foreign start-ups, after their entry	0.391***	8.9	125,247
Double difference (point estimate, F-test)	0.146**	9.5	

Significant at the **0.05, ***0.01 level. The t-values are based on standard errors adjusted for clustering by persons and firms

OLS regression with dummies standing for the four distinct groups. Dependent variable: log daily wage in the given month relative to the national mean. Sample: 471,489 person-months belonging to 8225 skilled workers hired by and staying until December 2011 in 519 newly established firms (366 domestic and 147 foreign-owned). We considered a firm newly established if its staff number jumped from less than 5 to more than 50, or, from less than 50 to more than 300 within a month. Workers employed by new firms before their ‘big bang’, workers leaving the new firms and firms changing owner after the big bang are excluded. Controls: person, job and firm characteristics and sector-year interactions. See Appendix 1: Table 12 for variable definitions

## Lagged returns

### Are ex-MNE workers paid more in the domestic sector?

In Eq. (2), we compare workers in domestic firms, who arrived at their employer from MNEs versus other domestic firms. The estimates are controlled for personal characteristics, current and past job attributes, tenure in the last job, months between the two jobs, selected indicators of the sending and receiving firms, and sector-year interactions. We retain firms with at least one ex-MNE and one ex-domestic employee and exclude firms undergoing acquisition.

3 $$\ln w_{ijt} = \alpha X_{it} + \beta_{1} F\_After_{ijt} + \beta_{2} dL_{jt } + \beta_{3} \left( {F\_After_{ijt} dL_{jt } } \right) + f_{j} + s_{jt} + \varepsilon_{ijt} .$$

$F\_After_{ijt}$ is a dummy set to 1 for workers who arrived from foreign firms and 0 for workers coming from domestic companies. $dL_{jt }$ = $L_{j,t + 1} /L_{j,t - 1}$ measures the change of employment in the sending firm between year $t{-}1$ and $t + 1$, with $t$ denoting the year when the worker left the firm. The coefficient $\beta_{2}$ measures how wages vary with employment dynamics of the sending domestic firms while the parameter $\beta_{3}$ of the interaction term $F\_After_{ijt}$ × $dL_{jt }$ captures the impact of d*L* on workers arriving from foreign employers. The wage advantage of workers coming from MNEs over workers arriving from domestic firms, conditional on employment dynamics of the sending firm, is given by $\beta_{1}$ + $\beta_{3} dL_{jt }$. Alternatively, we estimate the equation for two groups distinguished based on $dL$ (lower or higher than 0.5), without the size-change and interaction terms.

Since we are interested in the within-firm wage differences between ex-MNE and ex-domestic entrants (rather than how a worker’s wage changes upon entering a domestic firm), we include firm fixed effects, but not worker fixed effects.

The upper block of Table 5 shows the results of the first variant of the model. The wage advantage of an ex-MNE employee arriving from a firm where staff numbers did not change around the year of the worker’s separation ($dL$ = 1) amounts to 0.057 log points, while it is estimated to be 0.074 points in case the sending firm was closed or relocated ($dL$ = 0). We added a dummy indicating if the worker had arrived from another domestic firm but previously had some experience in one or more MNEs. These workers have an advantage of 0.064 log points. Only a part of these gaps results from within-firm premia, as suggested by the differences between the specifications with and without firm fixed effects.

**Table 5:** The wage advantage of ex-MNE workers in domestic firms over coworkers having arrived from other domestic firms—regression estimates

	Firm fixed effects:	
	No	Yes
Model A: entire sample		
Sending firm is MNE (F_After)	0.091*** (4.4)	0.077*** (4.9)
Change of employment in the sending firm (dL)	0.010* (1.8)	0.011*** (3.0)
Interaction term (F_After × dL)	− 0.028** (2.4)	− 0.024** (2.3)
MNE experience before entry to the sending firm (dummy)	0.059*** (6.0)	0.038*** (5.0)
Number of observations	723,421	722.913
aR^2^/within R^2^	0.461	0.288
Model B: workers arriving from mass layoffs and all workers		
Employment change in the sending firm: L_t+1_/L_t−1_ ≤ 0.5		
Sending firm is MNE	0.134*** (3.3)	0.109** (2.4)
MNE experience before entry to the sending firm (dummy)	0.068*** (2.9)	0.056*** (2.7)
Number of observations	153,482	153,213
aR^2^/within R^2^	0.479	0.277
Entire sample		
Sending firm is MNE	0.060*** (4.1)	0.049*** (5.1)
MNE experience before entry to the sending firm (dummy)	0.058*** (6.0)	0.037*** (5.0)
Number of observations	723,421	722,913
aR^2^/within R^2^	0.461	0.288

Significant at the *0.1, **0.05, ***0.01 level. The standard errors are adjusted for clustering by persons and firms. Sample: 723,421 person-months belonging to 96,277 skilled workers in 19,449 domestic firms, who had arrived from MNEs versus other domestic firms. 508 singleton observations are excluded from the equation with firm fixed effects. Estimation: Stata *reghdfe*. Change of employment in the sending firm: L_t+1_/L_t−1_, where *t* is the year of the worker’s separation. Controls: person and job controls, contemporaneous and lagged firm-level controls, as listed in Table 12. Additional controls are completed tenure in the sending firm, dummy for unobserved tenure, months between exit from the sending firm and entry to the receiving firm, one-digit sectoral affiliation of the sending and receiving firms and year dummies

The lower blocks of the table display estimates on two sub-samples distinguished along $dL$. Former MNE workers who lost or left their jobs during mass dismissals ($dL$ < 0.5) had substantially higher wage advantages over their ex-domestic counterparts (0.134 log points) than did those ex-MNE workers, who arrived from slightly contracting, stable or expanding firms (0.06).^19^

### An overlapping cohorts model of lagged returns to MNE experience

The estimates presented in the preceding sub-section are potentially subject to ability bias: workers returning to the domestic sector can be more productive wherever they work. As it was put forward in the Introduction, addressing this problem by adding worker fixed effects to model (2) is not a feasible option. Therefore, we estimate an alternative model that compares the wages of domestic firm employees with *past and future* experience in MNEs versus domestic companies other than their current employer. This approach is close in spirit to models that study the wage effect of incarceration by comparing past and future convicts (Grogger 1995; Pettit and Lyons 2009; LaLonde and Cho 2008; Czafit and Köllő 2015) under the assumption that the date of incarceration (mutatis mutandis the dates of entry to and exit from MNEs) can be treated as random. We can reasonably assume that future MNE workers are closer to former MNE employees in terms of unobserved characteristics than any control person selected from the general population based on observables. A further advantage of this choice is a gain in sample size: 3,841,561 person-months instead of 797,261 in Model (2).

We define a collectively exhaustive classification making a distinction between domestic firm employees with past MNE experience in month *t* (PF), workers with future but no past MNE experience (FF), workers with prior experience in other domestic firms and no MNE experience (PD) and workers with future domestic sector experience and none of the types mentioned earlier (FD). Incumbent workers who had no contact with other employers in 2003–2011 constitute the reference category. The sample we work with consists of domestic firm employees in companies employing at least one worker belonging to the categories mentioned above and one incumbent worker. We restrict the analysis to 2005–2009 to have sufficient observations on past and future experiences outside the workers’ current firms.

We regress log wages on the respective dummies and person, job, and firm-specific controls plus sector-year interactions. Choosing incumbents as the reference category and denoting the controls with **Z**, the estimated equation with or without firm fixed effects (*v*_j_) is:

4 $$\ln w_{ijt} = \beta_{1} PF_{ijt} + \beta_{2} PD_{ijt} + \beta_{3} FF_{ijt} + \beta_{4} FD_{ijt} + \left[ {v_{j} } \right] + Z\gamma + \varepsilon_{ijt} .$$

We measure the effect of foreign sector experience with the double difference ($\beta_{1}$ − $\beta_{2}$) − ($\beta_{3}$ − $\beta_{4}$) or equivalently ($\beta_{1}$ − $\beta_{3}$) − ($\beta_{2}$ − $\beta_{4}$). The model controls for unobserved differentials in worker quality as long as workers’ wages with future outside experience can be treated as a counterfactual for the wages of workers with prior experience. However, it cannot address the possibly endogenous selection of workers to separation from their previous employers.

The results in Table 6 show that workers with past MNE experience earn more by 0.112 log points than their counterparts with outside domestic experience. This difference overestimates the returns to foreign sector experience since those domestic workers who are on their way to an MNE also earn more by 0.043 log points than those about to leave for another domestic employer. Ex-MNE workers earn more than future MNE employees by 0.048 log points while those with outside domestic experience earn less by 0.021 log points than their counterparts, leaving for another domestic firm later.

**Table 6:** Wage difference between domestic workers with/without outside work experience

	Dependent variable: log daily wage	
	OLS	Firm fixed effects
Coefficients (t-test)		
Past MNE experience (PF)	0.060*** (4.0)	0.005 (1.0)
Future MNE experience (FF)	0.012 (0.9)	0.001 (0.6)
Past domestic experience (PD)	− 0.052*** (5.6)	− 0.030*** (7.3)
Future domestic experience (FD)	− 0.031*** (3.5)	− 0.016*** (3.6)
Differences by type of outside experience (F-test)		
Past MNE − past domestic	0.112*** (61.6)	0.035*** (53.4)
Future MNE − future domestic	0.043*** (15.0)	0.017** (5.5)
Past MNE − future MNE	0.048** (6.3)	0.004 (0.4)
Past domestic − future domestic	− 0.021** (4.2)	− 0.014*** (10.0)
Double difference	0.069*** (15.0)	0.018*** (11.5)
aR2/within R2	0.453	0.342

Regression estimates. The reported coefficients are significant at the *0.1, **0.05, ***0.01 level. Unmarked coefficients are not significant at the 0.1 level. Sample: 3,841,561 person-months belonging to 153,323 persons and 18,510 firms. The sample covers domestic firm employees in firms employing at least one worker with past or future experience in foreign-owned or domestic firms, and one incumbent worker. The coefficients measure wage advantages relative to incumbent workers. Observations for 2005–2009 are used. Estimation: reghdfe without and with firm fixed effects. The standard errors are adjusted for clustering by persons and firms. Controls: person, job and firm controls, and sector–year interactions

Using these estimates, we can approximate the return to MNE work experience as the double-difference equal to 0.069 log points. The two models’ main results aimed at measuring lagged wage effects (Tables 5 and 6) are similar. The first model identified a 0.060 log points advantage on the part of the median worker coming from an MNE over a worker arriving from domestic company (Table 5 bottom block).

While the main results are close to each other, some details differ in the two models. The wage difference between workers arriving from foreign-owned versus other domestic firms appear to be more prominent here: 0.112 points as opposed to 0.060 points in Table 5, model B, the estimate for all workers.^20^ Second, when we reestimate the model by adding firm fixed effects (column 2 of Table 6), the contrasts fade away: the within-firm wage differentials between the PF-FD groups are smaller, and the double-difference drops to only 0.018 log points. Unlike our first model, the second one suggests that the lagged MNE premium predominantly stems from past and future MNE employees’ crowding in high-wage domestic firms.

## Spillover effects

### Effect of ex-MNE peers on incumbent domestic firm employees

We estimate the effect of ex-MNE peers on incumbent workers’ wage, that is, for domestic firm employees who did not leave their firm in the observed period. Their wages are regressed on a set of controls and variables measuring the share of workers with previous outside experience within the worker’s company and skill category. We deviate from Poole (2013) in that we also study how skilled incumbents’ wages respond to the presence of less skilled ex-MNE peers. $Share_{jt}^{MNE,uskilled}$, for instance, measures the ratio of unskilled employees with recent MNE experience.

5 $$\begin{aligned} \ln w_{ijt} & = \theta_{F3} Share_{jt}^{MNE, skilled} + \theta_{F2} Share_{jt}^{MNE,middling} + \theta_{F1} Share_{jt}^{MNE,unskilled} \\ & \quad + \theta_{D3} Share_{jt}^{domestic,skilled} + \theta_{D2} Share_{jt}^{domestic, middling} + \theta_{D1} Share_{jt}^{domestic, unskilled} + \alpha X_{it} \\ & \quad + \beta Y_{ijt } + \gamma V_{jt} + v_{i} + s_{jt} + \varepsilon_{ijt} . \\ \end{aligned}$$

We estimate the model including only worker fixed effects, which also absorb the firm effects since the estimates relate to incumbent workers. The controls are identical to those used in Eq. 1. We restrict the time window to 2005–2011 to leave time for the accumulation of an ex-MNE stock. The equations are estimated separately for smaller (11–50) and larger (50+) firms, taking into consideration the higher risk of measurement error in small establishments.^21^

The fixed-effects panel equations summarized in Table 7 regress the log wages of incumbent skilled domestic workers on the share of workers with outside experience within the worker’s firm and skill group. The estimated own effect for skilled workers in a medium-sized or large firm ($\theta_{F3}$ = 0.074) implies that a one-standard-deviation difference in the share of high skilled ex-MNE employees (0.18) shifts the wages of skilled incumbents up by 1.3 percent. Having more skilled peers with outside experience in the domestic sector has no effect.

**Table 7:** The effect of coworkers with recent outside work experience on the wages of skilled incumbents in domestic firms 2005–2011

	Share of coworkers with recent MNE experience within skill groups			Share of coworkers with recent experience in other domestic firms within skill groups		
	Unskilled	Middling	Skilled	Unskilled	Middling	Skilled
Notations in Eq. 3:	θ_F1_	θ_F2_	θ_F3_	θ_D1_	θ_D2_	θ_D3_
All firms	0.012 (1.5)	0.003 (0.4)	0.042*** (3.9)	0.015** (3.1)	0.010 (1.5)	− 0.031*** (-4.5)
Firms employing > 50 workers	0.000 (0.0)	0.028 (1.2)	0.074*** (4.3)	0.005 (0.7)	0.042*** (2.8)	− 0.027** (− 2.1)

Significant at *0.1, **0.05, ***0.01 level. The t-values are based on standard errors adjusted for clustering by persons and firms.$\uptheta _{{{\text{F}}3}}$ is significantly larger than $\uptheta _{{{\text{F}}1}}$, $\uptheta _{{{\text{F}}2}}$ and $\uptheta _{{{\text{D}}3}}$. Sample: 3,730,789 person-months in 116,204 firms in the full sample, 2,474,830 person-months in 77,411 firms in the 50+ sample. Dependent variable: log daily wage in the given month relative to the national mean. Controls: person, job and firm characteristics, sector–year interactions, and worker fixed-effects

In evaluating the cross effects, one should consider the relevant range in the share of ex-MNE workers. While a jump from zero to 50 or 100 percent in the share of ex-foreign workers within the unskilled or medium-skilled workforce is beyond the realm of reality, which renders the spillover effect to be weak, this can happen in the high skilled category. Domestic firms employing 50 workers have 7 high skilled workers on average. Hiring two managers or professionals with foreign sector experience can increase the ex-MNE share from zero to almost 30 percent overnight, which implies a 0.022 log points wage increase for skilled incumbents.

### Reestimating spillover effects for all domestic firm employees

Incumbents in our data account for only 22 percent of the workers ever employed in the domestic sector and 34 percent of the workers never employed outside the domestic sector. The estimates of spillover effects using their sample may be biased because their exposure to peers with MNE experience differs substantially from that of the average worker. As shown in Table 8, the mean within-firm share of skilled MNE-experienced peers amounts to 9 percent in the case incumbents instead of 14.6 percent in the case of their non-incumbent counterparts—a predictable pattern since incumbents are more likely to be found in firms with low labor turnover.

**Table 8:** Mean within-firm share of coworkers with past MNE experience (percent)

	Skilled incumbents in domestic firms		Skilled domestic firm employees without MNE experience	
	Share of coworkers with MNE experience	Number of workers	Share of coworkers with MNE experience	Number of workers
Unskilled	7.0	38,355	13.3	73,320
Medium skilled	9.3	53,896	15.4	103,871
Skilled	9.0	55,900	14.6	107,250

Incumbents are workers, who had only a single domestic-owned employer in 2003–2011. The mean within-firm shares are weighted with firm size and relate to 2003–2011

A higher share of ex-MNE peers increases the likelihood of personal contacts, thereby assisting the diffusion of MNE-based skills within the firm. At the same time, the typical incumbent worker spends more time with the firm, so she has a better chance to absorb the imported knowledge. Because of the potential bias in either direction, we reestimate the spillover model for all domestic workers, including firm fixed effects on top of the worker fixed effects in the model to ensure that it identifies within-firm impacts.

The results for firms with more than 50 workers and all firms are presented in Table 9. Starting with the former: the own effect (0.060) is slightly lower than the point estimate for incumbents (0.074 in Table 4). Less skilled ex-MNE workers exert a weak effect—the respective coefficients are only significant at the 5 percent level. Having more skilled peers with recent outside experience in domestic firms do not affect wages positively at all. The estimates for all firms are much lower and insignificant at 5 percent level. The inward bias is probably explained by the noisy measurement of the F and D ratios in smaller enterprises.

**Table 9:** The effect of coworkers with recent outside work experience on the wages of skilled workers in domestic enterprises 2005–2011

	Share of coworkers with recent MNE experience by their level of skill			Share of coworkers with recent experience in other domestic firms by their level of skill		
	Unskilled	Middling	Skilled	Unskilled	Middling	Skilled
Notations in Eq. 3:	θ_F1_	θ_F2_	θ_F3_	θ_D1_	θ_D2_	θ_D3_
All domestic firms	0.007 (0.9)	0.013 (1.5)	0.020* (1.9)	0.016*** (3.2)	0.024*** (3.4)	− 0.037*** (− 4.5)
Domestic firms employing > 50 workers	0.006 (0.5)	0.057** (2.1)	0.060*** (3.4)	0.002 (0.2)	0.064*** (3.4)	− 0.019 (− 1.3)

Significant at *0.1, **0.05, ***0.01 level. The t-values are based on standard errors adjusted for clustering by persons and firms. $\theta_{F3}$ is significantly larger than $\theta_{F1}$ and $\theta_{D3}$, but not $\theta_{F2}$. $\theta_{F2}$ is significantly larger than $\theta_{F1}$

Sample: 3,731,548 person-months belonging to skilled workers in 116,249 firms in full sample, 2,474,843 person-months in 77,412 firms in the 50+ sample. Dependent variable: log daily wage in the given month relative to the national mean. Controls: person, job and firm characteristics, sector–year interactions, worker and firm fixed-effects

The estimated spillover effect might seem economically insignificant, but it is actually stronger than those we know from the literature. The study of Poole (2013)—which is closest to ours concerning method, sample characteristics, and industry coverage—estimated that at the average wage for a typical domestic worker, a 10 percentage points increase in the share of former MNE workers increased incumbents’ wages by $23 per year. This amount could buy a little more than one Starbucks solo espresso a month in Rio de Janeiro in 2015. The comparable estimate for skilled incumbents in our sample is $139 a year, which could buy 5.2 cups of Starbucks espresso a month in Budapest at 2015 prices.^22^

Learning from ex-MNE peers is only one explanation for the effect we identify. A firm’s effort to maintain its *wage ladder* after hiring a high-wage ex-MNE worker could occasionally motivate a firm to increase the wages of other employees. Still, we do not find this explanation convincing when spillover is observed in tens of thousands of firms. Why would so many domestic firms hire high-wage workers from MNEs if this decision implies further wage growth without an underlying rise in productivity? A positive selection of *all* workers to firms hiring from MNEs can also raise the average wage of coworkers with no MNE experience. However, our findings controlled for worker fixed effects and/or relating only to incumbents are free of this kind of bias. Last but not least, the finding that only skilled ex-MNE peers have an effect on skilled wages yields further support to the learning hypothesis.

## Two notes on differences by skills and sectors

**Fig. 1:**
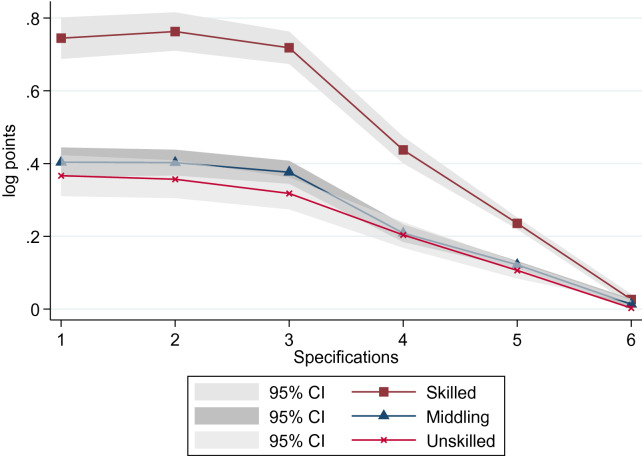
Estimates of the foreign-domestic wage gap by skills. Specifications: (1) sector–year interactions; (2) + person controls; (3) + job controls; (4) + firm controls; (5) + worker fixed effects; (6) + firm fixed effects. The confidence intervals are based on standard errors adjusted for clustering by persons and firms. On the sets of controls and the definition of skill levels, see Appendix 2: “Data”

**Fig. 3:**
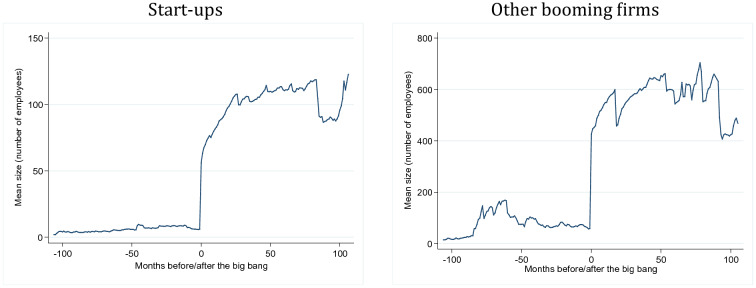
The mean size of firms classified as newly established. The data relate to 544 firms the size of which jumped from less than 5 to more than 50, or from less than 50 to more than 300 within a month (big bang). Firms changing majority owner after the big bang are excluded

Throughout this paper, we focused on skilled workers mainly because we are interested in possible knowledge flows from foreign-owned to domestic firms, the traces of which are easier to find in the skilled labor market.^23^ We nevertheless estimated all our models for less-skilled workers and found that the effects of interest are smaller and, in many cases statistically insignificant. Appendix 1: Fig. 1 illustrates this point. The figure compares the estimates of the wage gap model (Table 3, model A) to similar ones for unskilled and medium-skilled workers. The latter are very close to each other and amount to about 0.4 log points in the uncontrolled model, less than 0.1 in the panel regression with worker FE and less than 0.02 in the 2FE model.

Data available in the Labor Force Survey (Tables 13, 14 of Appendix 1) furthermore suggest that a part of the MNE premium compensates unskilled workers for non-wage disamenities. Overtime work and afternoon and night shifts are about twice as likely to occur among low and medium-skilled MNE employees compared to their domestic counterparts. There is a smaller but similarly signed difference concerning work on Saturdays and Sundays. Furthermore, low skilled workers have a higher probability of becoming unemployed in foreign-owned than domestic firms. The data does not indicate ownership-specific differences of this kind among highly skilled workers.

Table 10 summarizes point estimates of the wage gap, lagged returns, and spillover effects from our preferred model specifications for manufacturing and all other sectors labeled ‘services’. The foreign-domestic wage gap is more substantial in services than manufacturing, and the lagged returns are broadly similar or somewhat larger in services. By contrast, the spillover effects are estimated to be stronger in manufacturing. We do not go to the details of the between-sector differences. We only note that the returns to MNE experience are not restricted to the manufacturing sector heavily over-represented in the related literature.

**Table 10:** Selected estimates by sectors

	Manufacturing	Services
Contemporaneous MNE premium		
All firms, worker FE	0.152	0.236
New, incumbent firms, DiD	0.135	0.232
Lagged MNE premium in domestic firms		
Sending firm is MNE, dL < 0.5, OLS	0.135	0.133
Sending firm is MNE, dL < 0.5, firm FE	0.056	0.044
Overlapping cohorts estimate, DiD	0.027	0.072
Spillover effect, firms L > 50 employees		
On incumbents	0.088	0.057
On all workers with no MNE experience	0.069	0.050

All coefficients are significant at the 0.01 level. The coefficients were estimated separately for the two sectors

## Discussion

We interpret the coincidence of an MNE premium, substantial wage loss from separation, lagged returns to MNE experience, and wage spillover as a signal of knowledge flows from FDI to domestic firms. In such a scenario, workers acquiring general and firm-specific skills in the modern environment of MNEs are expected to earn more than their domestic counterparts. The specific components in their skills imply that MNE workers lose a part of their wage advantage in case of involuntary separation. The general component in their skills gives rise to wage advantages in their new, domestic firm and tends to influence their peers’ productivity. The simultaneity of these symptoms calls into question some alternative explanations, of which we discuss three ones.

First, the finding of a contemporaneous MNE premium even after controlling for worker fixed effects calls into question that the foreign-domestic wage gap is fully explained by the *crowding of high productivity workers* in foreign-owned firms. Similarly, in a comparison of domestic and foreign-owned start-ups, we find a sizable MNE premium even after controlling for their workers’ pre-entry wages.

Second, intense human capital accumulation is admittedly not the only potential source of an MNE premium, with the most important alternative being *efficiency wage setting*. MNEs may try to prevent leakage of information through labor turnover by paying a premium above the market level (Fosfuri et al. 2001). Their limited knowledge of the local labor market and capital-labor relations may urge them to pay high wages and share a part of their revenues with workers. Furthermore, they may try to compensate their employees for a higher labor demand volatility (Fabri et al. 2003) or a higher plant closure rate (Bernard and Sjoholm 2003). The implications of skills accumulation versus efficiency wages for the foreign-domestic wage gap and the wage loss from separation are observationally identical. However, efficiency wages in MNEs do not imply that ex-MNE employees earn a premium over the receiving domestic firm’s going wage rate and exert influence on the earnings of their peers.

Third, a set of findings like this is likely to emerge only if MNE workers accumulate both *general and firm-specific knowledge*. As outlined in Becker’s (1962) seminal paper, in the case of general skills acquired through on-the-job training, productivity and wages move in tandem. Workers accumulating a substantial stock of general skills in one firm are expected to earn higher-than-average wages in other firms. As far as general skills develop through informal communication between coworkers, their presence also tends to have a spillover effect. However, we do not expect that separation from an MNE induces a wage loss in this scenario.

If the acquired knowledge is purely firm-specific, and the risk of voluntary separation (motivated by factors other than between-firm wage differentials) is zero, then the firm pays the going market wage before, during and after the period of skills accumulation. These skills lose their value with separation without an impact on salaries. Pre-separation and post-separation wages are equal, post-separation wages do not exceed the host firm’s average level, and they do not exert influence on the earnings of coworkers. In the likely case of non-zero risk of voluntary quits, the firm will share in the costs and benefits of training, which implies lower wages in the accumulation phase and higher wages afterward as long as the worker stays with her employer. In this case, post-training involuntary separations imply a wage loss, but we continue not to expect lagged returns and spillover effects.

The literature emanating from Becker’s benchmark models has been trying to reconcile the theory of on-the-job training with a series of empirical observations inconsistent with the extreme scenarios. A series of empirical findings and ample everyday experience suggest that (i) most skills are general, or at least sector rather than firm-specific (ii) enterprises are willing to pay for general training, and (iii) involuntary separations typically imply a loss. Acemoglu and Pischke (1998) demonstrate that in a variety of market settings such as a compressed wage structure, substantial hiring costs, information asymmetry, and other labor market imperfections, general skills are rewarded as if they were partly specific. The “skill-weights” model of Lazear (2009) hypothesizes that skills are predominantly general, but firms attach different weights to their components. A worker who leaves a firm will have a difficult time finding another employer that can make use of all the skills he acquired at the sending firm. The limits of transferability impose a cost on mobile workers, so the workers are unwilling to bear the full cost of training, and the costs and benefits will be shared. Such a setting is likely to produce all of the four outcomes observed in our data.

## Conclusions

We found that high skilled MNE workers earn substantially higher wages than their domestic counterparts. They lose a part of their wage advantage after leaving the foreign-owned sector but, even so, they earn more than their domestic sector colleagues with no MNE experience. Their presence in domestic firms exerts a positive effect on the wages of their peers, who had no contact with foreign-owned firms or had no recent outside work experience at all.

The direct and indirect wage returns to work experience in MNEs are large in Hungary, similar to less developed countries analyzed in the literature. The positive wage effects are not restricted to the manufacturing sector, which is in the focus of attention in the research on FDI.The estimates suggest that the effect of MNE experience on domestic sector wages is strongly affected by between-firm variance, that is, the higher-than-average wages of domestic firms connected with the MNEs via labor turnover.

Finally, the results draw attention to the difficulties of identifying a ‘pure’ ownership effect. The non-random selection of firms flaws the identification of the foreign-domestic wage gap from acquisitions. Thanks to a rich and big data set, we could compare how workers are selected to new MNEs and domestic firms, and identify a substantial wage differential between them. In the analysis of lagged returns and spillovers, we drew attention to trade-offs between model quality and unbiasedness of the samples on which the models can be estimated.

As we find substantial wage effects attributed to foreign ownership both in the short-run and long-run, even after controlling for potential biases as much as possible, we believe that the presence and significance of knowledge transfer from MNEs is beyond doubt. Therefore, we argue that FDI coming from more developed countries exert positive effects on the receiving countries’ labor markets both through direct, and indirect channels. Exploring whether these gains outweigh the potential drawbacks could be the focus of future research on the topic.

## Figures and Tables

**Fig. 2:**
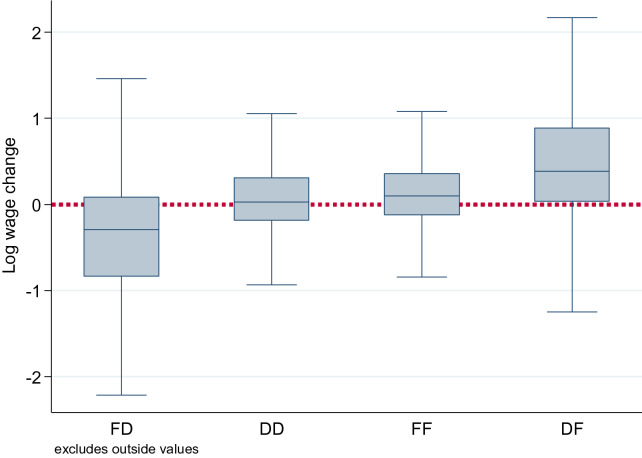
Shifts between sectors and wage change. The data relate to 307,874 shifts by skilled workers between ownership sectors in 2003–2011. F and D denote foreign-owned and domestic firms, respectively, in chronological order. The boxes display the interquartile ranges of log wage changes, with a horizontal line within the box indicating the median, and the whiskers showing the highest and lowest adjacent values. Heavy outliers are excluded. Wage change is measured as ln(w_1_/w_0_), where w_1_ and w_0_ are average earnings (normalized for the national mean) in the job spells after and before the shift, respectively

**Table 15:**
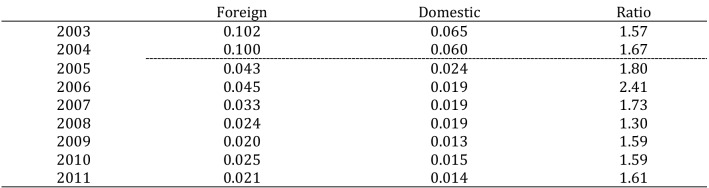
On-the-job training: fraction participating among MNE and domestic firm employees

High skilled employees working at least one hour in the reference week = 1

Source: Authors’ calculation using waves 45–80 of the LFS. Sample: ILO-OECD employed with college or university background. Key variables: participates in training of any kind outside the school system; the employer is majority or minority foreign-owned

Note that the question on participation changed in 2005. Figures above and below the dotted line are not directly comparable

## Data Availability

Due to the size and sensitivity of the data, access to it is provided exclusively for academic purposes by the Databank of the Centre for Economic and Regional Studies, Hungarian Academy of Sciences. We authorized the Databank to make the estimation samples and program codes available to referees (while maintaining their anonymity) and researchers willing to replicate the results. Access can be initiated at adatkeres@krtk.mta.hu.

## Appendix 1: Figures and tables

See Figs. 1, 2, 3 and Tables 11, 12, 13, 14 and 15.

**Table 11:** Descriptive statistics

	Mean	St. dev.
Male	0.619	
Age	37.9	10.4
Log health expenditures/national average wage	− 2.08	1.8
Receives disability pension/payment	0.006	
Receives care benefit	0.008	
Log regional unemployment rate	− 2.66	0.386
Central Hungary including Budapest	0.458	
Tenure is unobserved	0.398	
Tenure (months)	13.44	19.0
Top manager	0.051	
Other manager	0.271	
Professional	0.299	
Other white collar	0.112	
Skilled blue collar	0.025	
Assembler, machine operator	0.169	
Elementary occupation	0.012	
Agriculture	0.025	
Manufacturing	0.277	
Construction	0.061	
Trade	0.278	
Finance	0.126	
Energy	0.018	
Other services	0.215	
Foreign	0.397	
Domestic	0.603	
Firm size (log)	4.67	2.32
Fixed assets per worker (log)	7.92	1.81
Exporter	0.521	

Skilled workers, estimation sample for the wage gap model (Eq. 1)

Each variable covers 19,961,622 person months. The spells belong to workers employed at least once in a firm, the size of which exceeded the 10 workers limit at least once in 2003–2011. Public sector and state-owned firms are excluded. Note that other samples used in the paper have been drawn from this source file

**Table 12:** Pooled OLS results for Eq. 1, specification 4

	Coefficient	t-value
Majority owner^b^		
Foreign	0.437	20.4
Person controls		
Male	0.154	19.1
Age	0.032	15.2
Age squared/100	− 0.033	13.8
Months spent non-employed in 2003–2011	− 0.003	31.8
Receipt of disability payment	− 0.373	23.3
Receipt of care allowance	− 0.207	12.1
Health expenditures (log)	0.002	7.3
Job controls^d^		
Tenure if observed	0.001	4.3
Tenure is unobserved	0.138	12.2
Spell lasting for 1 day	0.354	3.1
Top manager	Ref.	
Other managers	− 0.062	1.6^n^
Professional	− 0.016	0.4^n^
Other white collar	− 0.298	9.0
Skilled blue collar	− 0.607	28.8
Assembler, machine operator	− 0.728	18.7
Laborer in elementary occupation	− 0.821	18.8
Regional unemployment rate (log)	− 0.063	5.2
Budapest	0.142	11.3
Firm controls		
Firm size (log)	0.086	7.3
Capital-labor ratio (log)	0.041	9.3
Exporter	0.185	9.4
Constant	− 1.650	24.0
Adjusted R-squared	0.479	
Number of observations	19,961,622	

Skilled workers, 2003–2011

Dependent variable: log daily earnings relative to the national mean. For the exact definition of the variables see Appendix 2: "Data". The coefficients of 63 sector–year dummies are not shown. The standard errors are adjusted for clustering by persons and firms. All coefficients are significant at 0.01 level except n) not significant at 0.1 level

**Table 13:** Incidence of atypical work schedules in foreign and domestic enterprises

Level of education^a^	Domestic	Foreign	Domestic	Foreign
	Shift work^b^		Overtime work^b^	
Low	27.5	58.2	14.4	32.0
Middling	22.4	41.2	12.0	24.3
High	4.4	4.5	4.0	7.6
	Work in the afternoon^c^		Work in the night^c^	
Low	14.4	29.1	8.1	20.3
Middling	18.6	33.1	9.4	22.1
High	17.7	14.4	7.1	6.6
	Work on Saturdays^c^		Work on Sundays^c^	
Low	26.3	29.3	16.9	17.6
Middling	35.4	36.6	21.2	24.0
High	26.8	18.9	16.7	12.7

2003–2011, percent

^a^Low = primary school attainment, High = college or university, Middling = rest

^b^Source: Wage Surveys, 2003–2011, private sector. Firms are classified on the basis of their majority owners. The data indicate the percentage share of employees receiving shift pay and overtime pay, respectively. Authors’ calculation

^c^Source: Labor Force Surveys, 2003 Q1–2011 Q4, excluding public administration, education, health and social services. The data indicate the percentage share of employees working in the respective periods at least occasionally. Authors’ calculation

**Table 14:** The effect of ownership on the probability of becoming unemployed—logit odds ratios

	Educational attainment^a^		
	Low	Middling	High
Employer: MNE	1.199*** (2.57)	0.971 (0.50)	1.061 (0.89)
Female	0.916 (1.40)	1.029 (0.55)	1.149*** (2.44)
Age	1.012 (0.71)	0.941*** (3.85)	0.919*** (5.01)
Age squared	0.999** (2.06)	1.000*** (3.08)	1.000*** (4.25)
Tenure (years)	0.894*** (9.27)	0.886*** (13.9)	0.895*** (10.0)
Number of observations	82,638	205,597	227,074
Pseudo R2	0.076	0.067	0.068
Wald chi^2^ (51)	617.4***	958.0***	763.8***

Significant at the **0.5 and ***0.01 level

Discrete time survival model, logit form, following (Jenkins 1995). Estimated for the pool of 28 quarterly waves of the Labor Force Survey in 2003–2009. The estimation excludes the crisis period (2010 and 2011). Sample: employees. Dependent variable: 1 if the person was ILO-OECD unemployed in wave *t* + 1 and 0 otherwise. The coefficients of 19 county dummies and 27 survey wave dummies are not shown

^a^Low = primary school attainment, High = college or university, Middling = rest

## Data

Starting sample: 50 percent random sample drawn from Social Security Numbers (SSN, Hungarian TAJ) valid on January 1, 2003. SSN holders aged 5–74 were retained. Data held by the Pension Directorate (ONYF), the Tax Office (NAV), the Health Insurance Fund (OEP), the Office of Education (OH), and the Public Employment Service (NMH) were merged and anonymized by the National Information Service (NISZ). The original data consisted of payment records with start and end dates, a type-of-payment code and amounts received by the person. Employers were identified by ONYF and their annual financial data were provided by NAV. The data was transformed to a fixed format monthly panel data set by the Databank of the Institute of Economics of the Hungarian Academy of Sciences.

Estimation sample: Workers employed with a labor contract at least once in a foreign or domestic private enterprise the maximum employment level of which exceeded the 10 workers limit at least once in 2003–2011. We removed workers and firms with less than two data points, zero wages and missing covariates. 98.5 percent of the workers belong to a single connected group.^24^ Special subsamples have been selected for the study of new firms, lagged returns and spillovers.

Data access: Data for the estimation samples and Stata do files are available on request. The original data set called Admin2 is also available via remote access to the Databank’s servers. Write to adatkeres@krtk.mta.hu for requesting access to the data. Note that the size of the original data ranges between 60 and 120 Gbytes, depending on the amount of information stored in special modules that you want to merge to the base file. The files are in Stata16 format. R and Python codes are allowed.

## Key variables

Wage: The daily wage figure used in the paper was calculated as monthly earnings divided by the number of days covered by pension insurance (‘working days’ henceforth) in the given month. Multiple payments made by the same employer to the same person within a month were summed up. Working days belonging to these payments were also summed up but capped at 30 or 31 days. In the case of multiple job holders the wage figure belongs to the highest paying job. We normalized the wage figures by dividing them with the national average wage in the given month, as measured in the starting sample. Source: ONYF.

Foreign-owned firm, MNE: dummy variable set to 1 for firms majority owned by one or more foreign owners. Ownership shares are measured as fractions of subscribed capital. Source: NAV.

## Person controls

Gender, age: Source: ONYF.

Skill levels: Skill levels are inferred from the ‘highest’ occupational status held by the person in 2003–2011. The classification is basedon one-digit occupational codes: 1 Top managers, 2 Other managers, 3 Professionals, 4 Other white collars, 5 Skilled blue collars, 6 Assemblers and machine operators, 7 Elementary occupations. Persons employed in occupations 1–3 at least once are classified as high skilled. Persons never employed outside occupations 6 and 7 are classified as low skilled. Other persons are classified as medium skilled. Source: ONYF.

Total time spent non-employed: The number of months out of employment in 2003–2011. Source: ONYF.

Disability payment: dummy variable, with 1 standing for any kind of transfer (pension or allowance) received on the basis of permanent disability (*rokkantnyugdíj*, *rokkantsági járadék*). Monthly data. Source: ONYF.

Care allowance: dummy variable, with 1 standing for any kind of benefit received by the observed person on the basis of raising children (*tgyás*, *gyed*, *gyes*, *gyet*) or taking care of disabled relatives (*ápolási segély*). Monthly data. Source: OEP, ONYF.

Health expenditures: Expenditures and costs registered by the National Health Insurance Fund (OEP). The items include total amount paid for OEP-supported medicine and the costs of OEP-supported services/treatment provided by district doctors, specialists and hospitals. We normalized the nominal figures by dividing them with the national average wage in the given month, as measured in the starting sample. Zero expenditures were replaced with 1 Ft (0.3 Euro cents per annum). Annual data. Source: OEP.

## Job controls

Tenure: Months elapsed since entry to the firm. Set to zero in the case of left-censored employment spells. A dummy stands for observations from left-censored spells.

Spell lasting for 1 day: Hungarian firms often pay to individual subcontractors by formally employing them on the day of payment. This practice results in exorbitant ‘daily wages’ in some cases.

Occupation: One-digit ISCO codes.

Regional unemployment rate: seasonally adjusted ILO-OECD unemployment rate in the given month and NUTS-2 region. The worker’s region is identified on the basis of his/her zip code in 2003. Source: author’s calculation using the Labor Force Survey.

## Firm controls

Firm size: average number of employees. Annual data. Source: NAV.

Capital-labor ratio: net value of fixed assets per worker. Annual data. Source: NAV.

Exporter: non-zero exports revenues. Annual data. Source: NAV.

Sector: NACE 2. Source: NAV.

## Footnotes

1 Section 2 provides a detailed introduction to the previous literature including the sources cited here.

2 MNEs may pay high wages to skim the cream of the labor force, buy loyalty, contain turnover, stimulate work effort, or prevent information leakage. Workers’ wages may fall upon leaving the MNE sector for losing these wage components and because employers perceive their dismissal as a negative signal. Ex-MNE workers may have high wages in domestic firms because they have high reservation wages and belong to the lucky few to find a well-paying job in the domestic sector. Spillover effects may arise from the employer’s wish to keep within-job wage differentials within tolerable limits.

3 We justify this choice and present some results on less skilled workers in Sect. 7.

4 Antalóczy and Sass (2001) estimate that the share of greenfield FDI in total inward FDI amounted to 25–30 percent in Hungary and other CEE countries during the transition.

5 With the requirement of controlling for lagged size changes, we would need workers with at least four employment spells in a nine-year-long period, with a specific pattern DDFD, where F and D stand for foreign-owned and domestic firms. Identification in this setting would come from comparing the second and fourth domestic job entries. The third, F spell is the treatment, and a first spell is required for the inclusion of firm sizes. Besides, in this setting the ex-MNE spells would sistematically happen later on in worker’s career, so life-cycle wage changes may be potentially captured by the parameter as well.

6 Which is a lower bound as in this model, we do not control for employment change in the sending firm.

7 Note that in the Norwegian case, workers moving from MNEs to domestic firms also acquire a gain of 7 percent, while in our sample they lose 11 percent. The median loss amounts to 26 percent in the case of skilled workers. See Table 2.

8 In the Czech Republic, Jurajda and Stančík (2012) detect sigificantly faster wage bill growth in (and only in) manufacturing firms with a low export share. They cannot decompose the wage bill effect into wage and employment effects.

9 They also show that domestic firms subject to foreign acquisition pay higher-than-average wages already before the takeover, hinting at a non-random selection to foreign buy-out.

10 See Appendix 2 for variable definitions.

11 Setting the limit elsewhere does not affect the results, since 93 percent of the firms with nonzero foreign presence are majority foreign-owned.

12 In 2014, MNEs had a 4.5 percent employment share in the 1–10 workers category (Authors’ calculation based on the 2014 Q4 wave of the Labor Force Survey).

13 This term hints at the practice of paying workers the minimum wage (subject to taxation) and the rest of their remuneration in cash. Elek et al. (2012) estimate that in 2006 the share of workers paid in this way amounted to 20 percent in firms employing 5–10 workers, 10 percent in slightly higher firms (11–20 workers) and less than 3 percent in larger enterprises.

14 UNECE (2001), p. 190.

15 See Appendix 1: Fig. 2 for a box-and-whiskers plot of wage changes.

16 The only exception would be observations on firms that, at the same time as changing ownership, would change all of their employees. We do not have such cases in the data.

17 Several methods have been developed in the last ten years (following the pioneering work of Abowd et al. 1999) to deal with two or more high dimensional fixed effects. The iterative methods (Cornelissen 2008; Martins and Opromolla 2009; Guimaraes and Portugal 2010; Carneiro et al. 2012; Mittag 2016) solve the problem by shuffling between the estimation of the slope and the intercept parameters. Balázsi et al. (2018) yield an alternative, which presses more on memory but runs faster. Early drafts of this paper, like Balázsi (2017) experimented with this method. With the size of the final data, iterative approaches turned out to be more productive.

18 Model B with added firm effects (Specification 6) is identical to Model B, as the always foreign indicator is absorbed by the added firm effects. Hence only a parameter on being temporarily foreign-owned can be estimated, which is identified only from firms going through acquisitions or divestments and accordingly, coincides with the parameter on the F dummy of Model A.

19 Workers who leave well-paying jobs in the MNE sector individually can be either negatively or positively selected. On the one hand, MNE employees fired individually are likely to be less productive than the average. On the other hand, those who manage to find a well-paid domestic job are predictably over-represented among voluntary quitters. The comparison of group-level estimates suggests that the first effect dominates: workers separating from their firms for reasons other than mass dismissals earn a lower lagged MNE premium on average.

20 The difference may stem from differences in the samples and the periods covered by the data as well as from the influence of experience in MNEs other than the sending firm. This effect is directly estimated in Table 5 but not in Table 6.

21 The fact that the Hungarian administrative panel is only a 50% sample on the individual level, has some unfortunate implications for the spillover estimates. We observe only around half of any given firm's labour force—estimates instead of the actual shares. As not observing ex-MNE workers has the same, 50% probability as those with no such experience, in large firms we will only experience extra noise in the share variables. This noise in our explanatory variable will attenuate the estimated $\theta_{es}$ parameters, biasing them towards zero. However, in firms with a small number of workers, if the average share of given type is also low, we may mistakenly not observe any variation in our variables of interest, while we should. If a firm which previously never had a foreign worker acquires a skilled manager with foreign experience, and we do not observe the given person, observations at this firm will not have variation in the share of skilled ex-MNE workers, thus this firm will not contribute to the identification of our parameter of interest in our model with firm fixed effects. Considering these two processes we not only keep solely the firms with at least 10 employees, as in most of the paper, but also focus on larger (50+ firms), where the (predicted) share variables are less volatile.

22 The calculation is based on the estimated own effect (0.074), the mean monthly earnings of skilled domestic firm employees in 2011 (236,078 Ft) and an average exchange rate of 225 Ft/$ in 2011 (National Bank, http://mnbkozeparfolyam.hu/arfolyam-2011.html). We could find Starbucks solo espresso prices for 2015 on the websites of local shops in Rio and Budapest: $1.92 and $1.43, respectively.

23 Skilled workers account for 25 percent of the total population observed in the source file. 15 per cent is unskilled (never worked in an occupation requiring nay kind of qualification) and 60 percent is classified as middling (worked in skilled jobs but not in ones requiring tertiary educational attainment).
